# Association of maternal pre-pregnancy body mass index with birth weight and preterm birth among singletons conceived after frozen-thawed embryo transfer

**DOI:** 10.1186/s12958-022-00957-8

**Published:** 2022-06-10

**Authors:** Jiaying Lin, Haiyan Guo, Bian Wang, Qianqian Zhu

**Affiliations:** Department of Assisted Reproduction, Shanghai Ninth People’s Hospital affiliated to JiaoTong University School of Medicine, Zhizaoju Road No. 639, Shanghai, China

**Keywords:** Body mass index, Large for gestational age, Preterm birth, Frozen embryo transfer

## Abstract

**Background:**

To explore the effect of pre-pregnancy body mass index (BMI) on neonatal outcomes among singletons born after frozen embryo transfer (FET).

**Methods:**

This large retrospective cohort study included 18,683 singleton infants born after FET during the period from Jan 1, 2007 to Dec 31, 2019. The main outcomes were large for gestational age (LGA) and preterm birth. Logistic regression models with generalized estimating equations for clustering by patients to estimate odds ratios of LGA and preterm birth.

**Results:**

Overweight was positively associated with LGA overall (adjusted OR 1.78 [95%CI 1.60-1.98]), and this association was consistent across age categories. The underweight was inversely associated with LGA among mothers younger than 35 years (adjusted OR 0.49 [95%CI 0.39-0.62] among mothers younger than 30 years; adjusted OR 0.47 [95%CI 0.37-0.60] among mothers aged 30-34 years), but this association was no significant among mothers 35 years or older. Overweight was positively and significantly associated with preterm birth overall (adjusted OR 1.52 [95%CI 1.30-1.77]) and consistently across age categories. The underweight mothers younger than 30 years had a decreased risk of preterm birth (adjusted OR 0.70 [95%CI 0.51-0.97]), but the underweight was no significantly associated with preterm birth among women aged 30 years of older.

**Conclusions:**

The risks of LGA and preterm birth were increased in singletons born to overweight mothers, regardless of the maternal age. Underweight decreased the risk of LGA and preterm birth for younger mothers. These findings are important for providing preconceptional counseling to specifically targeted women at high risk of LGA and preterm birth.

**Supplementary Information:**

The online version contains supplementary material available at 10.1186/s12958-022-00957-8.

## Background

Since the birth of the first in vitro fertilization (IVF) baby in 1978, more than seven million babies have been born worldwide through assisted reproductive technologies (ART). With the tremendous advancements in the field of ART over the past 40 years, the ultimate goal of ART has changed from achieving successful pregnancy to having a healthy baby [1]. In the recent decade, the improvement of embryo freezing technologies has significantly increased the number of frozen-thawed embryo transfer (FET) cycles. FET provided an optimized environment for the early developing embryo by transferring the thawed embryos in a natural cycle or an unstimulated artificial cycle, which helps to increase the pregnancy and live birth rates while decreasing perinatal and maternal morbidity [2, 3]. The increasing use of FET around the world has enhanced the safety awareness of this procedure.

The gestation age and birth weight are two important indicators evaluating neonatal health. Excessive fetal growth can result in birth weight of large for gestational age (LGA, birth weight > 90th percentile), which substantially increases the risk of short-term and long-term morbidities and mortality [4–7]. In the short term, LGA increases the difficulties in delivery, which can lead to hypoxic brain damage or even stillbirth [4]. In the long term, LGA infants are predisposed to developing obesity and type 2 diabetes in adulthood [5–7]. Preterm birth is defined as the delivery before 37 completed gestational weeks by the world Health Organization (WHO). It has been a critical global public health problem because of its leading cause for neonatal and childhood mortality and morbidity, and its association with the increased risk of neurodevelopmental impairments and chronic disease in adulthood for surviving preterm infants [8]. So clarify the influential factors of neonatal preterm birth and LGA, especially for singletons born after FET is important for public health to make substantive progress in decreasing the rates.

The extensive literature showed the increased risk of adverse neonatal outcomes in FET singletons comparing with singletons following spontaneous conception [9–11]. Many studies were performed to explore factors influencing neonatal risks in FET singletons, and mainly concerned about the reproductive technology per se or parental subfertility [9, 12, 13]. However, few researches about health outcome risks in FET children has focused on the effect of life style factors, such as maternal BMI, which were preventable by optimizing BMI of pre-pregnant women after professional pre-conceptional counseling.

Pre-pregnancy body mass index (BMI) is a potentially modifiable and preventable lifestyle-related factor related with neonatal outcomes [14]. However, the existing studies about the association of pre-pregnancy BMI and neonatal outcomes mainly focused on the babies born by natural conception, and the results were controversial and inconclusive [15]. Harsh et al. performed a systematic review and meta-analysis to explore the impact of maternal pre-pregnancy BMI on neonatal outcomes in the worldwide populations, and found overweight and obesity are related with the increased risk of LGA and underweight is related with the increased odds of SGA and preterm birth [16]. Rahman et al. conducted a systematic review and meta-analysis aiming to evaluate maternal BMI and risk of adverse perinatal outcomes in low- and middle-income countries, and also reported neonates of overweight and obese mothers did not have increased risk of preterm delivery and underweight mothers were at higher risk of SGA and preterm delivery [17]. However, another systematic review and meta-analysis was carried out by Liu et al. to establish the association of maternal BMI with neonatal outcomes in Chinese women, and reported the contrary result that overweight and obese women had an increased odd of preterm [18]. In addition, Liu et al. found infants of overweight or obese mothers were more likely to be LGA and pre-pregnancy underweight increased the risk of SGA. Very little research has been designed to explore the effect of pre-pregnancy BMI on neonatal outcomes among infants born after FET.

With the increasing use of FET worldwide, clinical study on the association of pre-pregnancy BMI and neonatal outcomes resulting from FET is very important to provide crucial advice to specifically target women at high risk of adverse neonatal outcomes before ART or during pregnancy. The objective of the current study was to assess the effect of pre-pregnancy BMI on adverse neonatal outcomes including LGA and preterm birth among singletons born after FET. Previous studies have reported the association between pre-pregnancy BMI and neonatal outcomes would differ by maternal age. A population-based cohort study including 7,141,630 singletons reported a crossover effect of maternal age (a change from an inverse association to a positive association) for the association between pre-pregnancy obesity and preterm birth [19]. Sever studies in teenage mothers reported the inverse association between pre-pregnancy obesity and preterm birth [20, 21]. So, we explore the relationship between pre-pregnancy BMI and neonatal outcomes across different age groups.

## Materials and methods

### Study design and data sources

All data used in this study were obtained from the ART database at the Department of Assisted Reproduction of the Shanghai Ninth People’s Hospital, affiliated with Jiao tong University, School of Medicine (a large hospital-based tertiary care reproductive center in Shanghai, China). Cycle-level information on patient characteristics, details of clinical treatment with ART procedure, and pregnancy outcomes and neonatal outcomes resulting from ART were recorded in this database, which was required by the Technical Standard for Human Assisted Reproduction issued by the Chinese Ministry of Health (CMOH) [22, 23]. Quality control methods were used to maintain a high-quality database. Trained medical staff carried out diagnosis verification, data checking and medical record review following standardized guidelines established by our center. Surveillance staffs randomly selected patients’ medical histories and verified data against the database. More than 60,000 frozen-thawed embryo transfer (FET) cycles were performed in our reproduction center since the initiation of our vitrification technique. In this study, we included patients who had a live singleton infant after vitrification-thawed embryo transfer during the period from Jan 1, 2007 to Dec 31, 2019 and available data for pre-pregnancy BMI, age, and gestational age and birth weight at birth. We excluded women who delivered a singleton after two fetuses were seen in the first trimester ultrasonographic examination. Patients with hypertension, diabetes, preeclampsia, or thyroid dysfunction were excluded, considering these diseases were strongly related with adverse neonatal outcomes. This study was approved by the Ethics Committee (Institutional Review Board) of the Shanghai Ninth People’s Hospital. This study was exempt from informed consent due to the retrospective nature and using anonymous clinical data.

### Procedures

Pre-pregnancy BMI was calculated as weight in kilograms divided by the square of height in meters. The weight and height were measured when the patient was prepared for the ovulation induction therapy. The pre-pregnancy BMI was categorized into three groups according to the world Health Organization classification: underweight (BMI < 18.5 kg/m^2^), normal weight (18.5-24.9 kg/m^2^), and overweight (25.0-29.9 kg/m^2^). In our population, the number of patients conforming to the WHO class I or higher obesity classes (BMI > 30 kg/m^2^) was small, so we did not analyze the data from these patients with a BMI > 30 kg/m^2^. The small number of obese women can be explained by the following reasons. The Asian population in general has a lower BMI than that observed for non-Asian populations [24]. In the other hand, weight loss is often be advised to obese infertile women before initiating ART cycles according to the clinical international recommendations [25]. The group with normal weight was used as the reference for the comparisons. For FET cycles, gestational age was calculated by adding 17 days for cleavage-stage embryo transfer and 19 days for blastocyst transfer from the date of embryo transfer. Large for gestational age (LGA) was defined as birth weight above the 90th percentile for gestational age, and small for gestational age (SGA) was defined as birth weight below the 10th percentile for gestational age according to the Chinese sex- and gestational age–specific birth weight standards [26]. Appropriate for gestational age (AGA) is the measure between the 10th and 90th percentiles for gestational age. Preterm birth was defined as the delivery before 37 completed gestational weeks by the world Health Organization (WHO).

### Statistical analysis

The distributions of basic characteristics in different BMI groups were calculated. Next, the BMI groups were stratified by maternal age, and the number and proportion of singleton births with SGA, AGA, LGA and preterm were examined in each group. Considering the repeated inclusion of 581 patients having two deliveries, thereafter, we used Logistic regression models with generalized estimating equations for clustering by patients to estimate unadjusted and adjusted odds ratios of LGA and preterm birth and corresponding 95% confidence intervals (CIs). Maternal age (< 30 years, 30-34 years, 35-37 years, and ≥ 38 years), infertility type (primary infertility and secondary infertility), parity (no child, one child, and two or more children), infertility diagnosis (tubal factor, ovulation dysfunction, diminished ovarian reserve, endometriosis, uterine factor, male factor, and unexplained or other factors), type of ART procedure (IVF, ICSI, mixed IVF and ICSI), number of embryos transferred (1 and 2), embryo stage at transfer (day 3 and day 5/6), infant gender (boy and girl), and year of birth were covariates adjusting for in analyses. Because the number of SGA babies was small especially for maternal age no less than 38 years and underweight or overweight which affected the accuracy of the result in multivariate analysis, so we did not present the result of SGA in the multivariate models in the tables. However, we have added the result bout the relationship between pre-pregnancy BMI and SGA in complementary material to avoid publication bias for future reviews and meta-analyses.

Stratified analyses by maternal age were used to assess potential disparities in the association between pre-pregnancy BMI and LGA or preterm birth. We also did analyses to evaluate the joint association of pre-pregnancy BMI and maternal age with risk of adverse neonatal outcomes. Two-tailed *P* values < 0.05 were considered significant. All statistical analyses were performed using the statistical package Stata, version 12 (StataCorp. Stata Statistical Software: Release 12. College Station, TX, USA).

## Results

A total of 18,683 live singletons born after FET were included in this study. Of these singletons, 2141 (11.46%) were delivered by mothers with underweight, 14,417 (77.17%) by mothers with normal weight, and 2125 (11.37%) by mothers with overweight. The basic characteristics across BMI groups are shown in Table 1. More than 30% of patients were younger than 30 years and more than 40% of patients were aged between 30 and 34 years in each BMI category. About half of patients were primary infertility and over 90% of patients had no child before ART across BMI groups. More than 70% of patients in each group were diagnosed as tubal factor infertility following by the male factor. IVF was the main fertilization method and about 80% of FET cycles were performed with two embryos and transferred embryos at day5/6 in all three groups.

**Table 1:** Characteristics of the frozen-thawed embryo transfer cycles by maternal pre-pregnancy body mass index

	Underweight, n (%) (BMI < 18.5 kg/m^2^)	Normal weight, n (%) (BMI 18.5-24.9 kg/m^2^)	Overweight, n (%) (BMI 25.0-29.9 kg/m^2^)
Overall	2141	14,417	2125
Maternal age (years)			
< 30	887 (41.43)^a^	4482 (31.09)^b^	664 (31.25)^b^
30-34	876 (40.92)	6281 (43.57)	903 (42.49)
35-37	282 (13.17)	2234 (15.50)	347 (16.33)
≥ 38	96 (4.48)	1420 (9.85)	211 (9.93)
Infertility type			
Primary infertility	1246 (58.20)^a^	7315 (50.74)^b^	1054 (49.60)^b^
Secondary infertility	895 (41.80)	7102 (49.26)	1071 (50.40)
Parity			
No child	2030 (94.82)^a^	13,227 (91.75)^b^	1921 (90.40)^b^
One child	106 (4.95)	1115 (7.73)	187 (8.80)
Two or more children	5 (0.23)	75 (0.52)	17 (0.80)
Infertility diagnosis^d^			
Tubal factor	1635 (76.37)^a,b^	11,223 (77.85)^a^	1587 (74.68)^b^
Ovulation dysfunction	128 (5.98)^a^	1364 (9.46)^b^	465 (21.88)^c^
Diminished ovarian reserve	81 (3.78)^a^	553 (3.84)^a^	47 (2.21)^a^
Endometriosis	278 (12.98)^a^	1447 (10.04)^b^	134 (6.31)^c^
Uterine factor	288 (13.45)^a^	2266 (15.72)^a^	319 (15.01)^a^
Male factor	1082 (50.54)^a^	7260 (50.36)^a^	1085 (51.06)^a^
Unexplained or others factors	81 (3.78)^a^	506 (3.51)^a^	72 (3.39)^a^
Type of ART procedure			
IVF	1346 (62.87)^a^	8994 (62.38)^a^	1294 (60.89)^a^
ICSI	560 (26.16)	3917 (27.17)	567 (26.68)
Mixed IVF and ICSI	235 (10.98)	1506 (10.45)	264 (12.42)
Number of embryos transferred			
1	397 (18.54)^a^	2631 (18.25)^a^	432 (20.33)^a^
2	1744 (81.46)	11,786 (81.75)	1693 (79.67)
Embryo stage at transfer			
Day 3	1747 (81.60)^a^	11,999 (83.23)^a^	1829 (86.07)^b^
Day 5/6	394 (18.40)	2418 (16.77)	296 (13.93)
Year of birth			
2007-2012	341 (15.93)^a^	1931 (13.39)^b^	235 (11.06)^c^
2013-2015	850 (39.70)	5602 (38.86)	753 (35.44)
2016-2019	950 (44.37)	6884 (47.75)	1137 (53.51)

*BMI* Body mass index body mass index (calculated as weight in kilograms divided by height in meters squared)

*ART* Assisted reproductive technology, *IVF* In-vitro fertilization, *ICSI* Intracytoplasmic sperm injection

^a,b,c^ the contrast between groups, and there is no statistical significance for the same letters

^d^More than one diagnosis per patient was possible

The proportion of singletons with SGA, AGA, LGA, and preterm birth across BMI categories stratifying by maternal age was presented in Table 2. Of mothers with pre-pregnancy underweight, the proportion of SGA was 7.71% (*n* = 165); mothers with normal weight had a similar proportion of SGA (4.29% [*n* = 618]) as mothers with overweight (4.24% [*n* = 90]). For mothers with underweight, 10.09% (*n* = 216) of singletons were LGA, whereas for mothers with normal weight, the percentage was 17.24% (*n* = 2486); for mothers with overweight, the proportion of LGA infant was 26.35% (*n* = 560). The proportion of preterm birth was 5.65% (*n* = 121), 7.38% (*n* = 1064), and 10.87% (231) for mothers with underweight, normal weight, or overweight, respectively.

**Table 2:** Offspring birth weight and preterm birth according to maternal age and pre-pregnancy body mass index

	Underweight, n (%) (BMI < 18.5 kg/m^2^)^*^	Normal weight, n (%) (BMI 18.5-24.9 kg/m^2^)	Overweight, n (%) (BMI 25.0-29.9 kg/m^2^)
SGA			
Overall	165 (7.71)	618 (4.29)	90 (4.24)
Age group, years			
< 30	62 (6.99)	193 (4.31)	27 (4.07)
30-34	71 (8.11)	254 (4.04)	42 (4.65)
35-37	23 (8.16)	109 (4.88)	14 (4.03)
≥ 38	9 (9.38)	62 (4.37)	7 (3.32)
AGA			
Overall	1760 (82.20)	11,313 (78.47)	1475 (69.41)
Age group, years			
< 30	736 (82.98)	3470 (77.42)	473 (71.23)
30-34	728 (83.11)	4952 (78.84)	617 (68.33)
35-37	219 (77.66)	1756 (78.60)	238 (68.59)
≥ 38	77 (80.21)	1135 (79.93)	147 (69.67)
LGA			
Overall	216 (10.09)	2486 (17.24)	560 (26.35)
Age group, years			
< 30	89 (10.03)	819 (18.27)	164 (24.70)
30-34	77 (8.79)	1075 (17.12)	244 (27.02)
35-37	40 (14.18)	369 (16.52)	95 (27.38)
≥ 38	10 (10.42)	223 (15.70)	57 (27.01)
Preterm			
Overall	121 (5.65)	1064 (7.38)	231 (10.87)
Age group, years			
< 30	47 (5.30)	322 (7.18)	66 (9.94)
30-34	50 (5.71)	441 (7.02)	97 (10.74)
35-37	19 (6.74)	175 (7.83)	40 (11.53)
≥ 38	5 (5.21)	126 (8.87)	28 (13.27)

*BMI* Body mass index (calculated as weight in kilograms divided by height in meters squared), *SGA* Small for gestational age (defined as birth weight below the 10th percentile for gestational age), *AGA* Appropriate for gestational age (the measure between the 10th and 90th percentiles for gestational age), *LGA* Large for gestational age (defined as birth weight above the 90th percentile for gestational age). Preterm birth was defined as delivery occurring before 37 weeks of gestation

Using the normal weight as the reference, overweight was positively associated with LGA overall (adjusted OR 1.78 [95%CI 1.60-1.98]), and this association was consistent across age categories, whereas the underweight was inversely associated with the risk of LGA among the overall mothers (adjusted OR 0.53 [95%CI 0.46-0.62]) (Table 3, Supplementary Material 1, Fig. 2). Statistically significant interaction was found between maternal pre-pregnancy BMI and maternal age on offspring LGA (p-interaction< 0.001). Stratification by maternal age and using birth to normal weight mothers as the reference, the underweight was inversely associated with LGA among mothers younger than 35 years (adjusted OR 0.49 [95%CI 0.39-0.62] among mothers younger than 30 years; adjusted OR 0.47 [95%CI 0.37-0.60] among mothers aged 30-34 years). To further assess the relationship between pre-pregnancy BMI and LGA, we evaluated the joint effects of maternal age with pre-pregnancy BMI on the risk of LGA (Supplementary Material 1, Fig. 1, Table 4). Mothers younger than 35-37 years had a lower risk of LGA than normal weight mothers aged 35-37 years (adjusted OR 0.59 [95%CI 0.46-0.75] among underweight mothers aged younger than 30 years; adjusted OR 0.50 [95%CI 0.39-0.65] among underweight mothers aged 30-34 years). For mothers who were overweight, the risk of LGA was significantly higher than normal weight mothers aged 35-37 years in all maternal age strata.

**Table 3:** Adjusted odds ratios (95%CI) for the relationship of pre-pregnancy body mass index with LGA and preterm, by maternal age

	LGA			Preterm		
	Underweight (BMI < 18.5 kg/m^2^)	Normal weight (BMI 18.5-24.9 kg/m^2^)	Overweight (BMI 25.0-29.9 kg/m^2^)	Underweight (BMI < 18.5 kg/m^2^)	Normal weight (BMI 18.5-24.9 kg/m^2^)	Overweight (BMI 25.0-29.9 kg/m^2^)
Overall^a^	0.53 (0.46,0.62)	Ref	1.78 (1.60,1.98)	0.76 (0.63,0.93)	Ref	1.52 (1.30,1.77)
Age group, years^b^						
< 30	0.49 (0.39,0.62)	Ref	1.56 (1.28,1.90)	0.70 (0.51,0.97)	Ref	1.45 (1.08,1.93)
30-34	0.47 (0.37,0.60)	Ref	1.83 (1.55,2.15)	0.79 (0.59,1.07)	Ref	1.56 (1.23,1.98)
35-37	0.84 (0.59,1.20)	Ref	1.98 (1.52,2.57)	0.88 (0.54,1.43)	Ref	1.54 (1.06,2.24)
≥ 38	0.62 (0.32,1.21)	Ref	2.01 (1.42,2.84)	0.58 (0.23,1.47)	Ref	1.48 (1.04,2.32)

*BMI* Body mass index (calculated as weight in kilograms divided by height in meters squared), *SGA* Small for gestational age (defined as birth weight below the 10th percentile for gestational age), *AGA* Appropriate for gestational age (the measure between the 10th and 90th percentiles for gestational age), *LGA* Large for gestational age (defined as birth weight above the 90th percentile for gestational age). Preterm birth was defined as delivery occurring before 37 weeks of gestation. Ref: reference

^a^Maternal age, primary infertility, parity, type of ART procedure, number of embryos transferred, embryo stage at transfer, infertility diagnosis (tubal factor, ovulation dysfunction, diminished ovarian reserve, endometriosis, uterine factor, male factor, unexplained or others factors), offspring gender, year of birth were adjusted for in models

^b^Maternal age was not included in this mode

All models included generalized estimating equations to account for clustering by patient

**Fig. 1:**
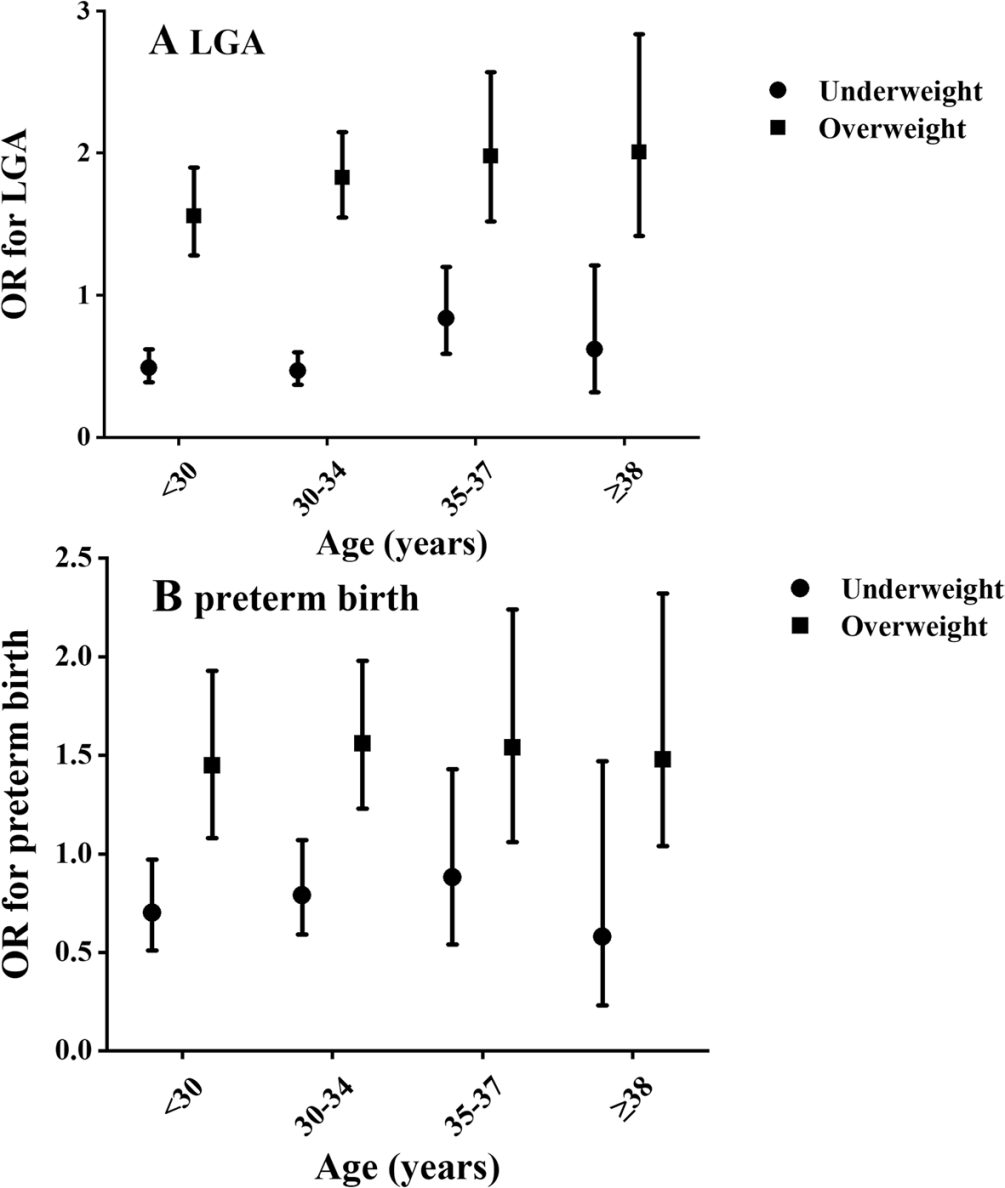
Association between pre-pregnancy BMI and risk of LGA (**A**) and preterm birth (**B**), by maternal age group

**Table 4:** Joint association of maternal age and pre-pregnancy body mass index with risk of LGA and preterm birth

	LGA			Preterm birth		
	Underweight (BMI < 18.5 kg/m^2^)	Normal weight (BMI 18.5-24.9 kg/m^2^)	Overweight (BMI 25.0-29.9 kg/m^2^)	Underweight (BMI < 18.5 kg/m^2^)	Normal weight (BMI 18.5-24.9 kg/m^2^)	Overweight (BMI 25.0-29.9 kg/m^2^)
Age group, years						
< 30	0.59 (0.46,0.75)	1.20 (1.05,1.38)	1.85 (1.49,2.29)	0.66 (0.47,0.92)	0.92 (0.76,1.12)	1.30 (0.96,1.76)
30-34	0.50 (0.39,0.65)	1.08 (0.95,1.23)	2.00 (1.66,2.42)	0.71 (0.51,0.99)	0.89 (0.74,1.07)	1.43 (1.09,1.86)
35-37	0.85 (0.60,1.22)	1.00(Ref)	1.95 (1.50,2.54)	0.86 (0.53,1.40)	1.00(Ref)	1.51 (1.05,2.18)
≥ 38	0.58 (0.30,1.13)	0.92 (0.77,1.11)	1.86 (1.34,2.58)	0.65 (0.26,1.61)	1.14 (0.90,1.45)	1.76 (1.15,2.71)

Women aged 35–37 years who were a normal weight before pregnancy were the reference group

Primary infertility, parity, type of ART procedure, number of embryos transferred, embryo stage at transfer, infertility diagnosis (tubal factor, ovulation dysfunction, diminished ovarian reserve, endometriosis, uterine factor, male factor, unexplained or others factors), offspring gender, year of birth were adjusted for in models

*BMI* Body mass index (calculated as weight in kilograms divided by height in meters squared), *LGA* Large for gestational age (defined as birth weight above the 90th percentile for gestational age). Preterm birth was defined as delivery occurring before 37 weeks of gestation

The model included generalized estimating equations to account for clustering by patient

In the overall study population, mothers with underweight had a significantly decreased risk of preterm birth compared with normal weight mothers (adjusted OR 0.76 [95%CI 0.63-0.93]), whereas overweight was positively and significantly associated with preterm birth overall (adjusted OR 1.52 [95%CI 1.30-1.77]) and consistently across age categories (Table 3, Supplementary Material 1, Fig. 2). Statistically significant interaction was found between maternal pre-pregnancy BMI and maternal age on offspring preterm birth (*p*-interaction< 0.001). Stratification by maternal age and using birth to normal weight mothers as the reference, the underweight mothers younger than 30 years had a decreased risk of preterm birth (adjusted OR 0.70 [95%CI 0.51-0.97]). To further assess the relationship between pre-pregnancy BMI and preterm birth, we evaluated the joint effects of maternal age with pre-pregnancy BMI on the risk of preterm birth (Supplementary Material 1, Fig. 1, Table 4). Compared with normal weight mothers aged 35-37 years, the risk of preterm birth decreased among mothers younger than 35-37 years (adjusted OR 0.66 [95%CI 0.47-0.92] among underweight mothers aged younger than 30 years; adjusted OR 0.71 [95%CI 0.51-0.99] among underweight mothers aged 30-34 years). For mothers who were overweight and older than 30 years, the risk of preterm birth significantly increased compared with normal weight mothers aged 35-37 years.

**Fig. 2:**
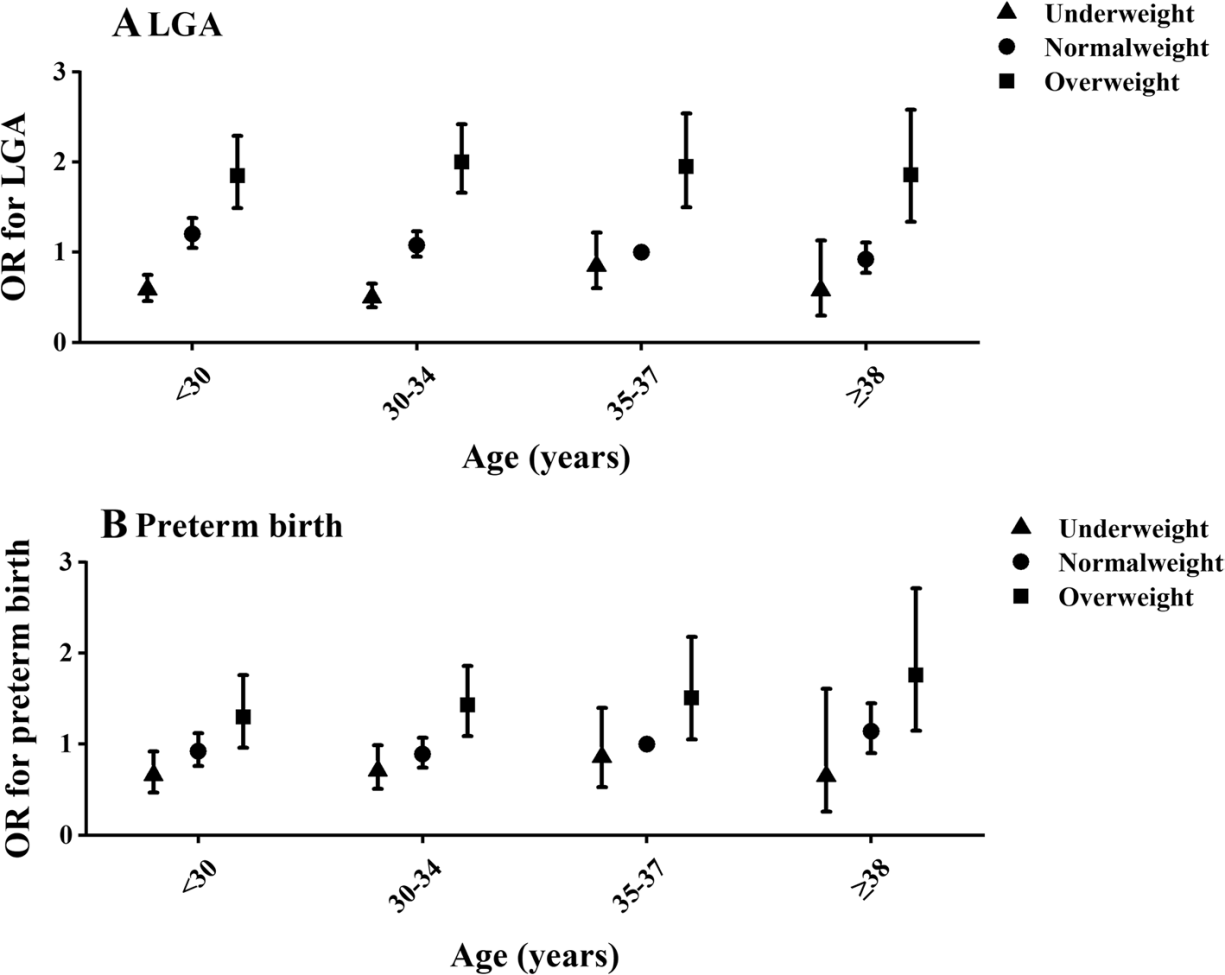
Joint association of maternal age and pre-pregnancy BMI with risk of LGA (**A**) and preterm birth (**B**)

## Discussion

### Principal findings

In this large observation study of more than eighteen thousand singletons born after frozen embryo transfer, we found that overweight women, irrespective of age, had significantly increased risk of LGA and preterm birth compared with normal weight women. Compared with normal weight women, a significantly decreased risk of LGA was found among underweight women younger than 35 years, and a significantly lower risk of preterm birth was found among underweight women aged less than 30 years. To our knowledge, this is the first study to explore the relation of pre-pregnancy BMI with the LGA or preterm birth, stratified by maternal age, among singletons born after FET.

### Strengths of the study

The primary strength of our study is that we focused on women conceived by FET. To our knowledge, this is the first and the largest study to explore the joint association of maternal pre-pregnancy BMI and age with the risk of LGA and preterm birth among FET offspring. Results from this study could enrich existing research on evaluating the effect of pre-pregnancy BMI on neonatal outcomes. The dataset was obtained from the single center, which reduced the potential confounding effects of differences in ART technique and laboratory environment between centers.

### Limitation of the data

However, several limitations need to be considered in interpreting our findings. First, although we had a large sample size, the number was small for subjects older than 38 years with underweight when we stratified by LGA or preterm birth. Second, we did not explore the association of obesity and preterm or LGA in this study owing to the small numbers of obese subjects. Third, as an observational study, we could not adjust for some potential confounders, such as endometrial preparation protocol, smoking status, a history of preterm delivery, nutrition conditions and educational status. Finally, patients with a BMI > 30 kg/m^2^ were not included in this study, which limited the generalizability to other populations with BMI > 30 kg/m^2^.

### Interpretation

The data from Global Burden of Disease Study showed the proportion of overweight has reached 38% globally for women aged 20 years or older [27]. In China, the percentage of overweight increased from 16.8% in 1992 to 26.4% in 2010 in women aged 18-44 years [28]. With the widespread epidemic of overweight, notably in developing countries, overweight have been recognized as a global public health issue [27]. Maternal pre-pregnancy overweight as a lifestyle-related factor already exceeded maternal smoking in its impact on neonatal health outcomes; many researches have been conducted to explore the relation of pre-pregnancy high BMI and neonatal birth weight [29]. Although pre-pregnancy overweight was associated with increased of LGA in some studies, in others no significant association between overweight and LGA was found. In a meta-analysis including 60 studies with 1,392,799 women, Liu et al. reported a significantly higher risk of LGA (OR 1.45, 95% CI: 1.29-1.63) among overweight mothers than normal weight mothers. However, more than half of the included studies in this research were from Southeast Asia or Africa, which limited the generalizability of the results to other population [14]. In another study of 2586 women in northern China, no significant association between overweight and LGA was found [30]. Our study, which exclusively focused on women undergoing FET, further enriched the current existing literature by suggesting that overweight exert an unfavorable effect on LGA for overweight women who were pregnant by FET. Results from several studies in recent years possibly explained the underlying mechanisms of this association between overweight and LGA. The dysregulation of glucose, insulin, lipid and amino acid metabolism probably play a certain role in the influence of maternal overweight and increased infant birth weight [31]. A mendelian randomization study including 30,487 newborns provided genetic evidence of causal relationship between elevated maternal BMI and higher offspring birth weight [32]. The potentially pathological and adaptive response of placenta to maternal increased BMI, including ultrastructural changes, accumulation of maternal macrophages, and increased placental weight, vascular muscularity and expression of inflammatory cytokines, may also help to explain the adverse neonatal health outcomes affected by overweight mothers [33–35].

Preterm birth is related with the high risk of neonatal mortality and morbidity [36]. Survivors of preterm birth are at increased risk of neurodevelopmental problems and long-term disability, which bring heavy financial and emotional burden to individuals, their families and countries [37]. So elucidating factors influencing preterm birth has important public health implication. Previous studies have been performed to explore the contribution of high pre-pregnancy BMI to preterm birth [15, 38, 39]. For example, McDonald et al. conducted a comprehensive systematic review of the studies published between 1950 and 2009 using a meta-analysis of 1,095,834 women from 84 studies from both developed countries and developing countries, and found that overweight women were significantly more like to have an infant of preterm birth [15]. However, to our knowledge, all previous studies did not specify whether the women were natural conception or assisted conception. Our study including 16,863 singletons born by FET found the risk of preterm birth was significantly increased in pre-pregnancy overweight women. This result suggested that the adverse effect of high pre-pregnancy BMI on neonatal preterm delivery was not overcome by FET. The underlying mechanism causing preterm birth may be explained by inflammation to some extent. Inflammation was proposed as an important risk factor of preterm delivery, and the inflammatory proteins (cytokines) including tumor necrosis factor (TNF) α, interleukin 6, and interleukin 1β increased in preterm birth [40]. High Maternal BMI could increase adipokines from adipose tissue and enhance systemic secretion of proinflammatory cytokines, which lead to the upregulation of the inflammatory pathway [41, 42]. Other mechanisms that may contribute to preterm birth in high BMI women include oxidative stress, insulin resistance, endothelial dysfunction, and lip toxicity [42, 43].

Although both developed and developing countries face a growing burden of overweight and obesity, underweight also remains a significant health public concern among women of childbearing age. By contrasting with previous studies finding that pre-pregnancy underweight was not related with LGA, our results suggest the pre-pregnancy underweight was a protective factor against LGA in women younger than 35 years [14]. However, the above association changes from positive to neutral for women aged 35 years or older. The exact underlying mechanisms that causing the relationship between underweight and LGA differ by age were poorly understood.

Existing evidences on whether lower maternal BMI influences the risk of preterm birth is debated. Han et al. performed a systematic and comprehensive summary of all studies between 1950 and 2009 including 78 studies and 1,025,794 women in developing and developed countries to elucidate the direction and magnitude of the relation between maternal underweight and preterm birth in singleton pregnancies [44]. The results from this study presented the positive association between preterm birth and underweight in developed countries (RR 1.22, 955CI 1.15-1.30), but no significant association was found in developing countries (RR 0.99, 955CI 0.67-1.45). For the study focused on specific population, Scholl and colleagues showed the underweight did not significantly affect the preterm birth in pregnant American adolescents [45]. Our study found an inverse association between underweight and preterm birth in women aged less than 30 years, but this association was insignificant in women 30 years or older. The genetic variation or difference in nutritional status may contribute to these disparities; however, further research is needed to verify these findings and to elucidate the underlying mechanisms.

## Conclusions

In this, the first and largest study evaluating the effects of pre-pregnancy BMI and maternal age on risks of LGA and preterm birth for singletons born after FET to our knowledge, to date, we observed an increased risk of LGA and preterm birth in singletons born to overweight mothers, regardless of the maternal age. Underweight decreased the risk of LGA and preterm birth for younger mothers. These findings are important for clinicians who provide pre-conceptional counseling to specifically targeted women at high risk of LGA and preterm birth. Overweight women should be informed of the perinatal risks and take some measures including dietary adjustment, physical exercise, and behavioral intervention, to optimize their BMI before fertility treatment.

## Supplementary Information

**Additional file 1: Supplementary table 1.** Odds ratios (95%CI) for the relationship of pre-pregnancy body mass index with SGA, by maternal age.

**Additional file 2: Supplementary table 2.** Joint association of maternal age and pre-pregnancy body mass index with risk of SGA.

## Data Availability

The datasets used and/or analyzed during the current study are available from the corresponding author on reasonable request.
