# Supplementary material for: Association of maternal pre-pregnancy body mass index with birth weight and preterm birth among singletons conceived after frozen-thawed embryo transfer
Source: Reprod Biol Endocrinol. 2022 Jun 10;20:86. doi: 10.1186/s12958-022-00957-8 (PMC9185967; doi:10.1186/s12958-022-00957-8)
Supplement: Supplementary file 1 — Additional file 1: Supplementary table 1. Odds ratios (95%CI) for the relationship of pre-pregnancy body mass index with SGA, by maternal age. [file 12958_2022_957_MOESM1_ESM.docx]

Supplementary table 1 Odds ratios (95%CI) for the relationship of pre-pregnancy body mass index with SGA, by maternal age

|  | SGA | | |
| --- | --- | --- | --- |
|  | Underweight  (BMI<18.5 kg/m^2^) | Normal weight  (BMI 18.5-24.9 kg/m^2^) | Overweight  (BMI 25.0-29.9 kg/m^2^) |
| Overall^#^ | 1.86(1.56,2.23) | Ref | 0.98(0.78,1.24) |
| Age group, years^*^ |  |  |  |
| <30 | 1.67(1.24,2.25) | Ref | 0.91(0.60,1.39) |
| 30-34 | 2.09(1.59,2.75) | Ref | 1.16(0.83,1.63) |
| 35-37 | 1.65(1.04,2.64) | Ref | 0.88(0.49,1.57) |
| ≥38 | 2.24(1.07,4.66) | Ref | 0.74(0.33,1.64) |

BMI: body mass index (calculated as weight in kilograms divided by height in meters squared); SGA: small for gestational age. Ref: reference.

^#^Primary infertility, parity, type of ART procedure, number of embryos transferred, embryo stage at transfer, infertility diagnosis (tubal factor, ovulation dysfunction, diminished ovarian reserve, endometriosis, uterine factor, male factor, unexplained or others factors), offspring gender, year of birth were adjusted for in models.

^*^Maternal age was not included in this mode.

All models included generalized estimating equations to account for clustering by patient.
