# Supplementary material for: Association of maternal pre-pregnancy body mass index with birth weight and preterm birth among singletons conceived after frozen-thawed embryo transfer
Source: Reprod Biol Endocrinol. 2022 Jun 10;20:86. doi: 10.1186/s12958-022-00957-8 (PMC9185967; doi:10.1186/s12958-022-00957-8)
Supplement: Supplementary file 2 — Additional file 2: Supplementary table 2. Joint association of maternal age and pre-pregnancy body mass index with risk of SGA. [file 12958_2022_957_MOESM2_ESM.docx]

Supplementary table 2 Joint association of maternal age and pre-pregnancy body mass index with risk of SGA

|  | SGA | | |
| --- | --- | --- | --- |
|  | Underweight  (BMI<18.5 kg/m^2^) | Normal weight  (BMI 18.5-24.9 kg/m^2^) | Overweight  (BMI 25.0-29.9 kg/m^2^) |
| Age group, years |  |  |  |
| <30 | 1.44(1.04,1.99) | 0.86(0.67,1.10) | 0.80(0.52,1.25) |
| 30-34 | 1.68(1.23,2.30) | 0.81(0.64,1.02) | 0.93(0.64,1.34) |
| 35-37 | 1.71(1.07,2.72) | 1.00(Ref) | 0.83(0.47,1.46) |
| ≥38 | 2.01(0.98,4.13) | 0.90(0.66,1.24) | 0.69(0.32,1.51) |

Women aged 35–37 years who were a normal weight before pregnancy were the reference group

Primary infertility, parity, type of ART procedure, number of embryos transferred, embryo stage at transfer, infertility diagnosis (tubal factor, ovulation dysfunction, diminished ovarian reserve, endometriosis, uterine factor, male factor, unexplained or others factors), offspring gender, year of birth were adjusted for in models.

BMI: body mass index (calculated as weight in kilograms divided by height in meters squared); SGA: small for gestational age.

The model included generalized estimating equations to account for clustering by patient.
